# New Method for Vulcanizing Rubber Plates

**Published:** 1890-11

**Authors:** 


### New Method of Vulcanizing Rubber Plates.

Dr. Geo. B. Snow has, by scientific and practical demonstrations, shown that a much better result can be obtained by a new method of vulcanizing rubber plates. "The *modus operandi* will be as follows: Pack the mould with rubber as usual, and close the flask either with bolts or a flask-press, as may be preferred. Before putting it in the vulcanizer slack off the bolts sufficiently to allow the flask to open when the rubber expands by heat, so that it will not be forced into the gateways. After it is vulcanized let it cool slowly until there is no steam pressure upon it; then remove the flask, place it in the spring clamp, replace it in the vulcanizer and re-heat it to 320°, and allow it to cool slowly. It is *always* best to keep the flask in the clamp and under pressure until cool.

In this case the mould contains more rubber than it would if held firmly, by the amount which would have been forced into the gateways by expansion as the heat was raised to the vulcanizing point; and this amount is sufficient, or nearly so, to completely fill the mould while hot and after vulcanization. The rubber is harder and not quite so tractable as when partly vulcanized, and the process requires more care and attention than is required with a vulcanizer capable of pressing the flask at the proper time while vulcanizing; but the operator is thus enabled to test the theory herein set forth, and to satisfy himself of the benefits to be secured by putting it into practice.

This process may be varied by first vulcanizing the plate about three-fourth the usual time; then applying the pressure and re-vulcanizing to finish.

The following precautions must be observed. Any sudden change in the steam pressure may result in the formation of steam in the flask, and injury to its contents; as it is not held together as it usually is. Therefore, no escape of steam by opening the blow-off, or blowing out the safety disk, should be allowed. Neither should the vulcanizer be suddenly cooled by putting it in water, or otherwise.

It is believed that the results obtained will amply repay the operator for what little additional trouble he will incur in using this method of vulcanizing."

